# Effects of Ketamine on Resting-State EEG Activity and Their Relationship to Perceptual/Dissociative Symptoms in Healthy Humans

**DOI:** 10.3389/fphar.2016.00348

**Published:** 2016-09-27

**Authors:** Sara de la Salle, Joelle Choueiry, Dhrasti Shah, Hayley Bowers, Judy McIntosh, Vadim Ilivitsky, Verner Knott

**Affiliations:** ^1^School of Psychology, University of OttawaOttawa, ON, Canada; ^2^Department of Cellular and Molecular Medicine, University of OttawaOttawa, ON, Canada; ^3^Department of Psychology, University of GuelphGuelph, ON, Canada; ^4^University of Ottawa Institute of Mental Health ResearchOttawa, ON, Canada; ^5^Department of Psychiatry, University of OttawaOttawa, ON, Canada; ^6^Royal Ottawa Mental Health CentreOttawa, ON, Canada

**Keywords:** ketamine, *N*-methyl-D-aspartate, brain oscillations, electroencephalography, psychosis, schizophrenia

## Abstract

*N*-methyl-D-aspartate (NMDA) receptor antagonists administered to healthy humans results in schizophrenia-like symptoms, which preclinical research suggests are due to glutamatergically altered brain oscillations. Here, we examined resting-state electroencephalographic activity in 21 healthy volunteers assessed in a placebo-controlled, double-blind, randomized study involving administration of either a saline infusion or a sub-anesthetic dose of ketamine, an NMDA receptor antagonist. Frequency-specific current source density (CSD) was assessed at sensor-level and source-level using eLORETA within regions of interest of a triple network model of schizophrenia (this model posits a dysfunctional switching between large-scale Default Mode and Central Executive networks by the monitor-controlling Salience Network). These CSDs were measured in each session along with subjective symptoms as indexed with the Clinician Administered Dissociative States Scale. Ketamine-induced CSD reductions in slow (delta/theta and alpha) and increases in fast (gamma) frequencies at scalp electrode sites were paralleled by frequency-specific CSD changes in the Default Mode, Central Executive, and Salience networks. Subjective symptoms scores were increased with ketamine and ratings of depersonalization in particular were associated with alpha CSD reductions in general and in specific regions of interest in each of the three networks. These results tentatively support the hypothesis that pathological brain oscillations associated with hypofunctional NMDA receptor activity may contribute to the emergence of the perceptual/dissociate symptoms of schizophrenia.

## Introduction

Blockade of glutamate *N*-methyl-D-aspartate receptors (NMDAR) by a subanesthetic dose of ketamine in humans transiently induces negative, positive and cognitive symptoms similar to those found in schizophrenia (SZ). Together with findings in animals of behavioral, biochemical, and electrophysiological alterations with NMDAR antagonists, convergent genetic, molecular and neuroimaging evidence points to NMDAR hypofunction in SZ (Javitt, 2012; Javitt et al., 2012). This supports use of the ketamine challenge as a pharmacological approach to generating insights into SZ symptoms and their underlying pathophysiologic processes (Frohlich and Van Horn, 2014).

Oscillatory brain activity is considered a fundamental process in the temporal coordination of circuits linked to perceptual, cognitive, and emotional functions. This brain activity, as measured by the amplitude/power of electroencephalographic (EEG) rhythms, is increasingly seen as an indicator of intrinsic brain function when assessed during stimulation/task-free states (Spencer, 2014). Further, this activity has been found to be altered in SZ and to vary with symptomatology (Boutros et al., 2008, 2014; Moran and Hong, 2011; Galderisi et al., 2014). Aberrant “resting-state” spectral EEG profiles in psychosis have invariably shown increased activity in low frequency (delta, theta) EEG and magnetoencephalographic (MEG) rhythms (Fehr et al., 2003; Boutros et al., 2008; Venables et al., 2009; Uhlhaas and Singer, 2010; Moran and Hong, 2011; Ranlund et al., 2014) which have been found specifically evidenced in chronic SZ patients (Sponheim et al., 1994, 2000; Narayanan et al., 2014), their first-degree relatives (Narayanan et al., 2014) and first-episode SZ patients (Sponheim et al., 2000), but not in individuals at-risk for psychosis (Ranlund et al., 2014). Spontaneous alpha activity, which predominates in healthy individual’s EEG, is significantly diminished in chronic SZ patients (though unaffected in some studies), with varying findings that depend on scalp region, and whether slower or faster frequencies are examined within this band (Sponheim et al., 1994, 2000; Knyazeva et al., 2008; Hong et al., 2012; Narayanan et al., 2014; Goldstein et al., 2015; Kim et al., 2015). Similar inconsistencies are evident with beta rhythms, with chronic patients showing either no abnormalities (Wada et al., 1994; Kam et al., 2013; Kim et al., 2015) or increased activity (Sponheim et al., 1994; Venables et al., 2009; Narayanan et al., 2014). As with delta/theta findings (Galderisi et al., 2009), these inconsistencies can be partially attributed to the effects of chronicity, medication and patient symptomatology (Czobor and Volovka, 1992; Merlo et al., 1998; Knott et al., 2001; Cerdán et al., 2005; Venables et al., 2009; Moran and Hong, 2011). The recent interest in resting gamma activity, increased in some (Czobor and Volovka, 1992; Merlo et al., 1998; Knott et al., 2001; Cerdán et al., 2005; Kam et al., 2013; Kocsis et al., 2013; Tikka et al., 2013, 2014, 2015) but not all EEG or MEG studies (Sponheim et al., 1994) of SZ patients (Rutter et al., 2009; Fuggetta et al., 2014; Hirano et al., 2015) is of particular interest both because of its purported role in feature binding and coordination of local neuronal populations, and because these rhythms are in part dependent on the activity of Parvalbumin-containing GABAergic cortical inhibitory interneurons, which are diminished in SZ patients and subject to NMDAR modulation from excitatory pyramidal cells (Lee et al., 2003; Herrmann and Dermirlap, 2005; Sun et al., 2011; Gandal et al., 2012; Lewis et al., 2012; Cohen et al., 2015). Integrative brain functions, however, are modulated through multiple oscillatory processes in different frequency bands, each with differing neuropharmacological sensitivities. As such, the study of the full frequency spectrum and its pharmacological basis is critical for deciphering the complex electrophysiologic abnormalities in SZ patients (Moran and Hong, 2011).

Electrophysiological recordings in animals with NMDAR antagonists such as ketamine and MK-801 recreate some of the background spontaneous EEG features of SZ (Hunt and Kasicki, 2013). Consistent with patient findings, increases in delta are dose-dependent and evident in cortical and subcortical (hippocampus, thalamus) regions. Theta frequency activity also shows these dose related changes, with power in this band consistently increased in cortex but reduced in the hippocampus. Ketamine’s effects on these rhythms in rodents, although generally augmenting beta and gamma by its actions on PV-interneurons in multiple cortical regions (Pinault, 2008; Carlén et al., 2012), vary depending on dose, acute vs chronic treatments, subcortical region and within-band (slow vs. fast) frequencies (Pinault, 2008; Roopun et al., 2008; Hakami et al., 2009; Carlén et al., 2012; Kittelberger et al., 2012; Hunt and Kasicki, 2013; Kocsis et al., 2013). In humans administered relatively high (anesthetic) doses of ketamine (~2 mg/kg), activity is increased in theta and beta bands and decreased in alpha (Schultz et al., 1990; Bojak et al., 2013). Sub-anesthetic psychotomimetic bolus doses of ketamine (0.2–0.5 mg/kg) have also increased theta and decreased alpha in baseline (pre-stimulus) EEGs (Kochs et al., 1996). In the only two human resting-state EEG studies to date, reductions in delta, alpha, and beta (gamma not assessed) were found with an ultra-low ketamine dose (Knott et al., 2006), and increases in gamma (other bands not assessed) were observed with a psychotogenic dose, but relationships with induced dissociative or psychotic-like symptoms were not examined (Sanacora et al., 2014). Of the two human resting MEG studies, subanesthetic ketamine increased gamma in both while reducing either alpha (Muthukumaraswamy et al., 2015) or beta (Rivolta et al., 2015).

Ketamine-modulated neuroelectric activity in rodent models suggest complex-spatiotemporal effects, which vary with frequency band and cortical area (Lee et al., 2003; Herrmann and Dermirlap, 2005; Pinault, 2008; Roopun et al., 2008; Moran and Hong, 2011; Carlén et al., 2012; Gandal et al., 2012; Lewis et al., 2012; Hunt and Kasicki, 2013; Cohen et al., 2015). Similar systematic EEG studies on healthy humans of the effects of subanesthetic, psychotomimetic doses of ketamine on the generation and cortical distribution of intrinsic brain rhythms across the frequency spectrum have not been conducted. Thus, one of our objectives has been to profile the acute effects of ketamine on the generation and topography of sensor-level EEG in low and high frequencies using reference-free current source density (CSD) measures (Nunez and Srinivasan, 2006). Such reference-free measures avoid problems associated with reference-dependent EEG indices (Kayser and Teake, 2010); reference-independent CSD measures reduce volume conduction from distal sites, sharpen spatial resolution, and are more closely related to neuronal activity and the strength of underlying current generators (Nicholson, 1973; Nunez and Srinivasan, 2006; Kayser and Teake, 2010).

A second objective has been the evaluation of the effects of NMDAR blockade on source-level, electric activity within Nekovarova et al.’s (2014) recent triple network (TN) model of SZ psychopathology. Largely investigated with fMRI (Fox and Raichle, 2007), but also assessed with PET (Raichle et al., 2001) and EEG (Chen et al., 2008; Yuan et al., 2012), brain regions have been shown to be dynamically organized into functional networks of intercorrelated areas (or “nodes”) that act together to perform specific tasks (Bressler, 1995). Of the full repertoire of brain networks, all of which are continuously and dynamically “active” not only during cognition but even when at “rest” (Smith et al., 2009), during sleep (Fukunaga et al., 2006) and under anesthesia (Vincent et al., 2007), there are three large-scale domain-specific networks that make up the model which exhibits abnormalities in SZ (Bressler and Menon, 2010) and depression (Mulders et al., 2015): the default mode network (DMN), the central executive network (CEN), and the salience network (SN). This TN model relies on accumulating neuroimaging evidence in healthy humans indicating that SN, which is involved in the orientating of attention to the most homeostatically relevant (salient) of ongoing extrapersonal (sensory) and intrapersonal (limbic driven) events (Bressler and Menon, 2010), causally influences anticorrelated activation of DMN and CEN. Clinical and cognitive symptoms of SZ are in part attributed to aberrant switching by the SN between internal processes (i.e., self-referential, autobiographical functions) supported by the DMN and external processes (i.e., attention and processing of exogenous stimuli) supported by the CEN as a result of a dysfunctional SN, a system that enables the switch between various dynamic brain states (Sridharan et al., 2008; Palaniyappan et al., 2012).

Adopting a region of interest (ROI) approach with exact low resolution brain electromagnetic tomography (i.e., eLORETA), which is an electrophysiological neuroimaging method that allows a reliable source localization of surface level electrical signals (Pascual-Marqui, 2011; Pascual-Marqui et al., 2011), we assessed ketamine-induced changes in two key regional nodes anchoring each of the three large-scale networks: DMN [ventromedial prefrontal cortex (VMPFC) and posterior cingulate cortex (PCC)]; CEN [dorsolateral prefrontal cortex (DLPFC) and posterior parietal cortex (PPC)]; and SN [anterior cingulate cortex (ACC) and anterior insula (AI)]. Based on limited human EEG studies with NMDAR antagonists, we hypothesized that relative to placebo, ketamine administration in healthy humans would produce SZ-like scalp surface CSD changes (↑ delta, beta and gamma, and ↓ alpha). Ketamine has been shown to alter resting SN and DMN function (Bonhomme et al., 2016) and given the pivotal role of the SN in the TN model and the available neuroimaging evidence indicating a primary role of dopamine in the interaction of the SN with subcortical sites, whilst the within-network activity of the SN and its interaction with other large-scale networks is thought to predominantly depend on glutamate/gamma-aminobutyric acid (GABA) neurotransmission (Palaniyappan et al., 2012), we expected ketamine to result in band-dependent CSD alterations within the cortical networks. We further hypothesized that these sensor- and source-level EEG changes would be accompanied by dissociative symptoms characteristic of SZ.

## Materials and Methods

### Participants

A sample of 21 right-hand dominant, male volunteers (mean age = 21.3 years, ±2.5 SE) were recruited via local media advertisements. Male volunteers were chosen in order to avoid any potential confounding effects of menstrually related hormonal changes on ketamine response. Volunteers underwent both a psychiatric interview, using both the Structured Clinical Interview, non-Patient version (SCID-NP) for DMS-IV (Williams et al., 1992) and the Family Interview for Genetic Studies (FIGS), (Maxwell, 1992) and a medical exam, including electrocardiogram and routine blood/urine laboratory tests as well as using toxicology for drug use. Only healthy individuals who were medication-free, non-smokers (smoked < 100 cigarettes, none in the past year), reported no neurological disease, and had no personal or family (first-degree biological relatives) psychiatric/substance abuse history were included in the study. The protocol was approved by the Research Ethics Board of the Royal Ottawa Health Care Group and all participants provided written informed consent. This study was conducted in accordance with the Tri-Council Policy Statement on Ethical Conduct for Research Involving Humans. Participants were compensated $75 CAD for each of the two test sessions.

### Design

Volunteers participated in the test sessions within a randomized, double-blind design in which half were administered placebo in the first session and ketamine in the second session, while the remaining half received treatments in the reverse order. A minimum 5 days interval separated the two test sessions.

### Procedures

Test sessions (beginning 8:00 a.m.) followed overnight abstinence from food, caffeine, alcohol, and drugs and began with insertion of an antecubital intravenous line after which, participants rested for a 45-min adaptation period and EEG electrodes were positioned on the scalp. A racemic ketamine or saline 0.90% w/v of NaCl bolus dose was then administered (0.26 mg/kg) over 10 min and was immediately followed by a constant infusion of 0.65 mg/kg lasting ~60 min, as per Krystal et al. (1999). Beginning 10 min after the initiation of the constant infusion, a 3 min EEG recording was collected, after which participants were evaluated with the Clinician Administered Dissociative States Scale (CADSS), (Bremner et al., 1998) which has been previously shown to be sensitive to ketamine administration (Krystal et al., 1994). The scale consists of 19 self-report items, (scored as 0 = not at all; 1 = slightly; 2 = moderately; 3 = considerably; 4 = extremely), which yielded three subjective subscale scores (amnesia, depersonalization, and derealisation) and it also has similarly scored 8 clinician rated items that result in one observer rated score.

#### EEG Recording

Electroencephalographic activity was recorded according to recommended pharmaco-EEG standards (Knott, 2000; Saletu et al., 2006; Jobert et al., 2012) while participants were seated reclined with eyes closed. Using an electronically linked mastoid reference, Brain Vision^®^ Recorder software Version 2 (Brain Products, Munich, Germany) was used to sample (1000 Hz) electrical activity from 28 EEG and 2 (vertical and horizontal) electrooculograpic (EOG) channels, with amplifiers/filters set at 0.1–100 Hz, and with electrode impedance < 5 kΩ. The electrodes were divided into nine regions, including left frontal (Fp1, F7, F3) middle frontal (Fz, FC1, FC2), right frontal (Fp2, F4, F8), left temporal-central (T7, FC5, CP5), middle temporal-central (C3, Cz, C4), right temporal-central (T8, FC6, CP6), left posterior (P7, P3, O1), middle posterior (Pz, CP1, CP2), right posterior (P4, P8, O2).

#### Scalp Surface CSD Analysis

Off-line processing with Brain Vision^®^ Analyzer 2 software (Brain Products, Munich, Germany) involved: visual inspection of recordings for elimination of activity with prominent ocular/muscle/cardiac contamination or drowsiness (i.e., alpha suppression combined with increased slow waves), ocular correction of EEG with an algorithm (Gratton et al., 1983); automatic rejection of activity with voltages > 100 uV and finally application of independent component analysis (ICA) to remove residual ocular (e.g., microsaccades) artifacts (McMenamin et al., 2010). The resulting artifact-free recordings (minimum 120 s) were transformed into reference-free CSD estimates using a spherical Laplacian algorithm (Nunez et al., 1997; Mima and Hallett, 1999). CSD estimates were computed using the fourth order spherical spline interpolation, and a maximal degree of Legendre polynomials of 10 (Perrin et al., 1989). Corrected, non-overlapping 2-s epochs were subjected to a Fast Fourier Transform algorithm (using a Hanning window with 10% taper length) for computation of absolute CSD (averaged across epochs at each electrode site) in delta (1–4 Hz), theta (4–8 Hz), alpha (8–12.5 Hz), beta (12.5–30 Hz), and gamma (30–60 Hz) bands. A natural log transform was applied to computed CSD values (Gasser et al., 1982) and individual electrodes were aggregated to create an average CSD value for anterior, temporal-central, and posterior regions.

#### Source-Localized CSD Analysis

eLORETA (version 2081104) was used to compute the intracortical source distribution of the electric activity from the surface EEG data (Pascual-Marqui, 2011; Pascual-Marqui et al., 2011). eLORETA is a weighted minimum non-linear inverse solution method applied to EEG recordings for computation of three dimensional distribution of electric cortical activity with zero location error (Pascual-Marqui, 2011; Pascual-Marqui et al., 2011). Localization with this methodology, even with a lower number of electrodes used in this study has been cross-validated with functional and structural MRI, PET and intracranial recordings. Relying on the Montreal Neurologic Institute average MRI brain (MNI 152) (Canuet et al., 2011) and a solution space restricted to cortical gray matter/hippocampus, eLORETA analysis of each EEG epoch results in current density being computed at each of 6239 cortical voxels (5 mm spatial resolution) for each of the frequency bands. Results were averaged across epochs for each individual and frequency band in placebo and ketamine sessions. Defined ROIs were based on definitions of the Brodmann Areas (BA) provided by eLORETA software package, which are based on the Talairach Daemon^1^. A single voxel (at the centroid of each BA) was used for each ROI due to eLORETA’s restricted spatial resolution, which makes it unable to separate two closely spaced sources, and additionally, the single centroid voxel (the closest to the center of the BA mass) is an excellent representative of the corresponding BA. The BAs comprising the representative hubs of the three targeted networks within the TN model included: BA 11 (VMPFC), BA 23/30 (PCC), BA 9 (DLPFC), BA 40 (PPC), BA 24/32 (ACC), and BA 47 (AI). CSDs were derived for left and right hemisphere of each BA.

### Statistics

Statistical analysis was conducted in SPSS 23 (SPSS Inc., Chicago, IL, USA). For each frequency band, scalp CSD values were analyzed with a separate repeated measures analysis of variance (ANOVA) involving treatment (placebo, ketamine), region (anterior, temporal-central, posterior) and laterality (left, middle, right) factors. Separate repeated measures ANOVAs were also conducted for each band-indexed network and involved treatment, hemisphere (left, right) and network hub (two levels) factors. Significant (*p* < 0.05) Greenhouse-Geisser estimates were followed up with Bonferroni adjusted *T*-test comparisons. The three primary rating measures, depersonalization, derealisation and observation, evidenced non-normal distributions and were analyzed by the non-parametric Wilcoxon Signed Ranks Test (WSRT). Relationships were examined with ketamine-induced CSD and CADSS difference scores, obtained by subtracting values in the placebo session from values in the ketamine session, and analysis with the non-parametric Spearman’s correlation coefficient. For source-localized EEG, these CADSS-CSD relationships were separately examined for each hub of each network. For scalp EEG, changes in CADSS were examined in relation to the CSD averaged across the ketamine affected electrode sites.

## Results

### CADSS Rating Scores

**FIGURE 1:**
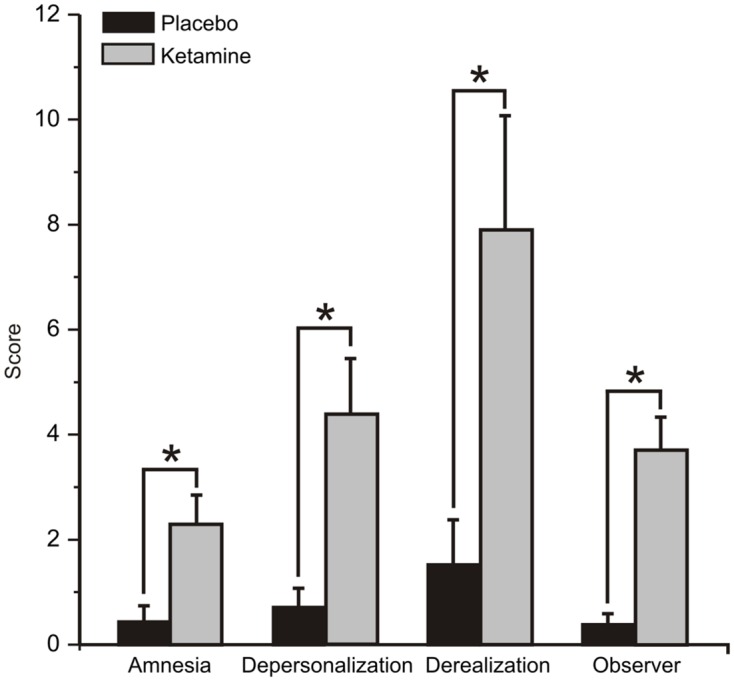
**Mean (±SE) Clinician Administered Dissociative States Scale (CADSS) values for the rating scores for the placebo and ketamine conditions (*n* = 21).** ^∗^*p* < 0.05.

The mean (±SE) values for the rating scores for the placebo and ketamine conditions are displayed in **Figure 1**. Significant increased self-ratings for amnesia (WSRT = -2.83, df = 1/40, *p* < 0.005), depersonalization (WSRT = -3.53, df = 1/40, *p* < 0.0001), and derealisation symptoms (WSRT = -3.36, df = 1/40, *p* < 0.01) were shown for the ketamine compared to placebo infusion condition. Ketamine also increased the observer rated symptom scores (WSRT = -3.72, df = 1/40, *p* < 0.0001) relative to placebo.

### Scalp Surface CSD

**FIGURE 2:**
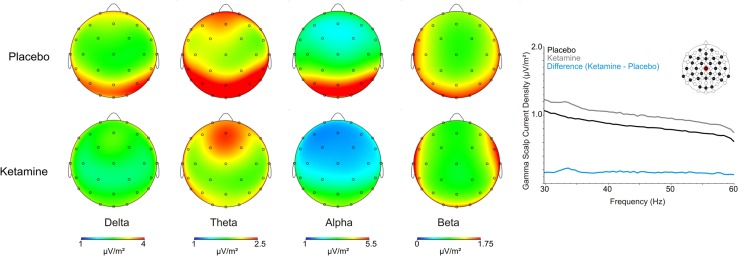
**Grand averaged scalp current source density (CSD) topographic maps for the placebo and ketamine conditions for the frequency bands, together with a spectral graph of gamma from the Cz site**.

As shown in **Figure 2**, scalp analyzed current density values for slow and fast oscillations were significantly influenced by ketamine. Delta exhibited significant treatment (*F* = 6.14, df = 1/20, *p* < 0.02) and treatment × region interaction effects (*F* = 3.52, df = 2/40, *p* < 0.04), with ketamine acting to reduce CSD bilaterally in temporal-central (*p* < 0.02) and posterior (*p* < 0.01) regions. Within a treatment region × laterality interaction (*F* = 3.77, df = 4/80, *p* < 0.007), theta CSD was similarly reduced in the left (*p* < 0.04) and right (*p* < 0.03) posterior cortex. Exhibiting treatment (*F* = 9.91, df = 1/40, *p* < 0.005), treatment x region (*F* = 4.43, df = 2/40, *p* < 0.02) and treatment × region × laterality interaction effects (*F* = 4.35, df = 4/80, *p* < 0.003), alpha current density was significantly diminished in left (*p* < 0.002) and right (*p* < 0.01) anterior, middle (*p* < 0.006) and right (*p* < 0.004) temporal-central, and posterior regions following ketamine infusion. Beta current density was not affected by ketamine treatment but significant treatment effects for gamma (*F* = 5.49, df = 1/20, *p* < 0.03) showed that ketamine increased current density of these oscillations across all scalp regions. **Table 1** displays a summary of the regional effects of ketamine vs. placebo.

**Table 1:** Summary of significant ketamine-induced scalp surface current source density (CSD) regional changes associated with each frequency band.

Delta	Theta	Alpha	Beta	Gamma
↓ Left, Right temporal-central	↓ Left, Right Posterior	↓ Left, Right Anterior	No changes	↑ Left, Middle, Right Anterior
↓ Left, Right Posterior		↓ Middle, Right temporal-central		↑ Left, Middle, Right temporal-central
		↓ Left, Middle, Right Posterior		↑ Left, Middle, Right Posterior

### Source-Localized CSD

#### Default Mode Network (DMN)

**FIGURE 3:**
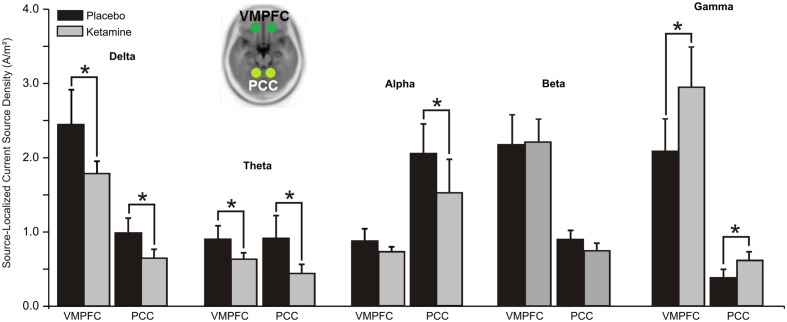
**Mean (±SE) non-logged source-localized CSD values (A/m^2^) for the two regions of interest of the default mode network (*n* = 21).** ^∗^*p* < 0.05. VMPFC, ventromedial prefrontal cortex; PCC, posterior cingulate cortex.

Significant ketamine effects on ROIs of the DMN are shown in **Figure 3**. Analysis of delta yielded a significant treatment × hemisphere interaction (*F* = 6.93, df = 1/20, *p* < 0.007), with ketamine (vs. placebo) acting to reduce CSD in the right hemisphere of the VMPFC and PCC (*p* < 0.05). A significant treatment effect (*F* = 5.46, df = 1/20, *p* < 0.03) showed general ketamine-induced reductions in theta CSD across both hubs of the DMN. Within treatment (*F* = 12.49, df = 1/20, *p* < 0.004) and treatment × hub interaction effects (*F* = 5.58, df = 1/20, *p* < 0.03), ketamine was found to diminish alpha CSD in the PCC (*p* < 0.002).

In contrast with the other oscillatory bands, gamma CSD was significantly increased by ketamine in both the VMPFC and PCC hubs of the DMN (*F* = 5.72, df = 1/20, *p* < 0.03).

#### Central Executive Network (CEN)

**FIGURE 4:**
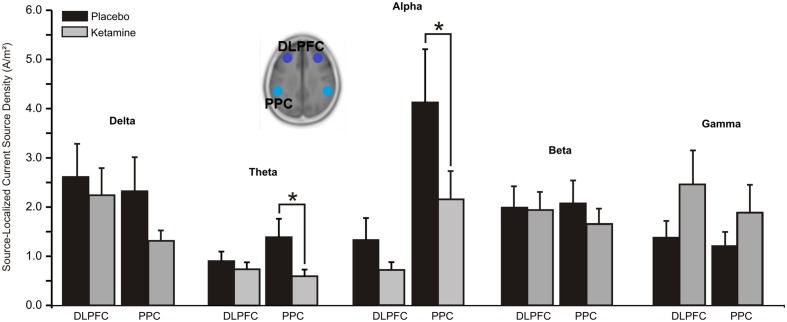
**Mean (±SE) non-logged source-localized CSD values (A/m^2^) for the two regions of interest of the central executive network (*n* = 21).** ^∗^*p* < 0.05. DLPFC, dorsolateral prefrontal cortex; PPC, posterior parietal cortex.

For both theta (*F* = 7.21, df = 1/20, *p* < 0.01) and alpha (*F* = 6.03, df = 1/20, *p* < 0.02), significant treatment × region interactions saw the CSD of these oscillations in the PPC to be reduced by ketamine compared to placebo (**Figure 4**). No significant treatment effects were observed for delta, beta or gamma CSD.

#### Salience Network (SN)

**FIGURE 5:**
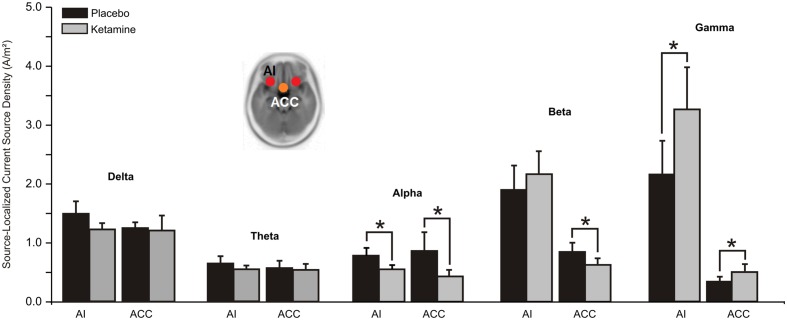
**Mean (±SE) non-logged source-localized CSD values (A/m^2^) for the two regions of interest of the salience network (*n* = 21).** ^∗^*p* < 0.05. AI, anterior insula; ACC, anterior cingulate cortex.

Whereas a significant overall treatment effect (*F* = 7.96, df = 1/20, *p* < 0.01) showed alpha CSD to be reduced in both ACC and AI hubs of the SN, a treatment x region interaction evidenced with beta (*F* = 10.47, df = 1/20, *p* < 0.004) found current density reductions to be limited to the ACC (*p* < 0.05). Ketamine, by contrast, significantly (*F* = 4.85, df = 1/20, *p* < 0.04) increased ACC and AI gamma current density (**Figure 5**).

### CADSS-CSD Relationships

**FIGURE 6:**
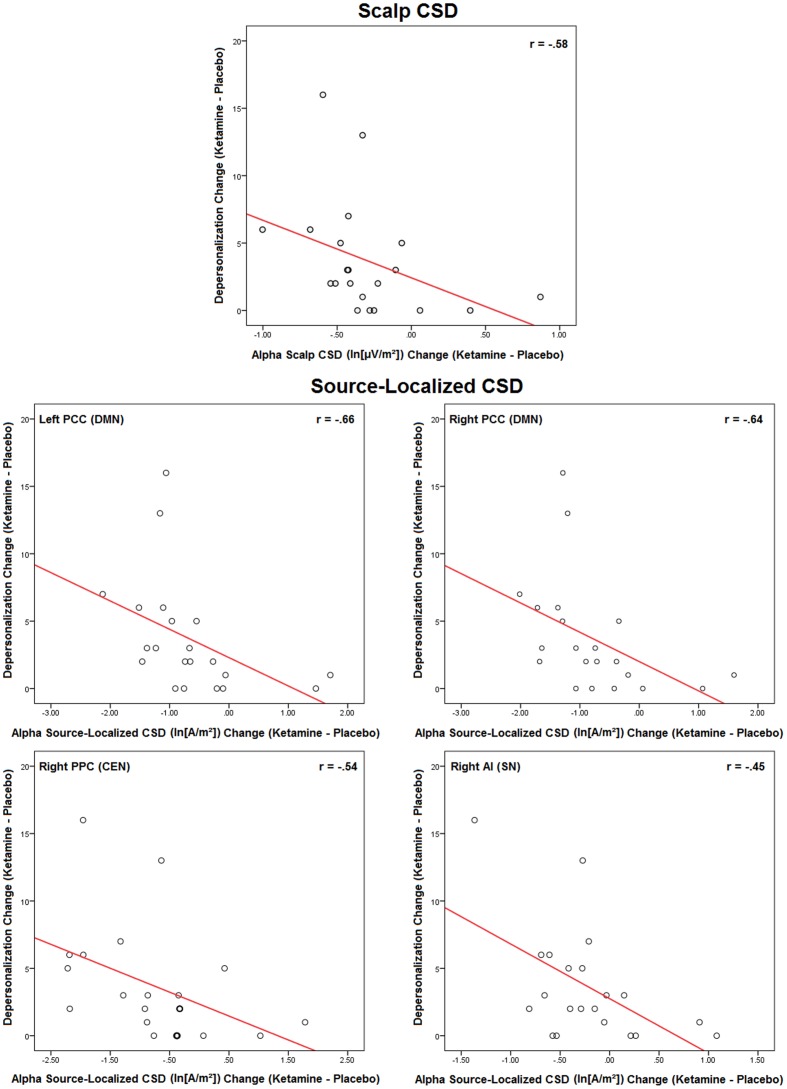
**Scatterplots of significant ketamine-induced changes in scalp and source-localized EEG and changes in CADSS scores induced by ketamine (*n* = 21).** Scalp CSD scatterplots represent averaged values of significant regional changes.

Changes in symptoms induced by ketamine were significantly associated with ketamine-induced changes in scalp and source-localized EEG, but only with alpha CSD (**Figure 6**). Scalp EEG alpha changes were negatively correlated with depersonalization ratings (*r* = -0.58, *p* < 0.006). For source-localized EEG, reductions in alpha CSD in the left (*r* = -0.66, *p* < 0.04) and right (*r* = -0.64, *p* < 0.002) PCC hemispheres of the DMN, and the right PPC (*r* = -0.54, *p* < 0.01) and AI hemispheres (*r* = -0.45, *p* < 0.04) of the CEN and SN, respectively, were related to increased depersonalization rating scores. No other correlations were evidences with delta, theta, beta, or gamma rhythms.

## Discussion

Although there are plenty of single blind EEG/MEG studies, this is the first randomized, placebo-controlled, double-blind crossover study of healthy humans administered a sub-anesthetic, psychotomimetic dose of ketamine that demonstrates changes in resting-state EEG across the frequency spectrum. The work builds on extensive animal research on the electrophysiological effects of ketamine and well-studied EEG correlates of anesthetic doses of ketamine. As key findings, we report CSD alterations in low and high frequency rhythms analyzed from scalp EEG recordings and these were accompanied by similar current density changes in source-localized cortical ROIs within the three large-scale networks of the schizophrenia model, namely the DMN, CEN and SN. These electrocortical effects of ketamine were paralleled by changes in dissociative states, the degree of which was significantly correlated with scalp and SZ network alpha current density changes.

*N*-methyl-D-aspartate receptor antagonism in preclinical models has most frequently been associated with increases in low (delta/theta) and high (beta/gamma) frequencies (Hunt and Kasicki, 2013). These *in vivo* electrocortical findings with NMDA receptor blockade, paralleled to some extent by oscillatory changes in *in vitro* (Kim et al., 2015) recordings and intracortical networks (Hakami et al., 2009), vary considerably, particularly with respect to theta rhythms, which have also been shown to be reduced both with acute and chronic ketamine treatment (Kittelberger et al., 2012). Our EEG observations in healthy volunteers of increased gamma and reduced non-gamma activity with a ketamine dose typically used to model SZ in part confirm previous independent study findings of EEG alpha and gamma changes seen with anesthetic and sub-anesthetic dosing in humans (Schultz et al., 1990; Kochs et al., 1996; Knott et al., 2006; Bojak et al., 2013; Sanacora et al., 2014) and they correspond with ketamine-induced alpha/beta and gamma alterations in recent resting MEG studies (Muthukumaraswamy et al., 2015; Rivolta et al., 2015). These emerging electrocortical patterns with NMDA receptor blockade seen in our study and in other investigations are reminiscent of some of the reports of aberrant EEG activity in SZ. Deficits in resting spontaneous alpha band activity reported across the clinical course of the disease (chronic SZ, first episode psychosis, prodromal SZ, relatives of SZ probrands) (Boutros et al., 2008; Goldstein et al., 2015; Kim et al., 2015) were mimicked in our EEG recordings across scalp regions following NMDA antagonist treatment. They were also seen in recent MEG recordings (Muthukumaraswamy et al., 2015), and were concurrent with diffuse increments in gamma also described in some recent resting state studies of SZ (Kam et al., 2013; Kocsis et al., 2013; Tikka et al., 2013, 2014, 2015). These parallel observations with alpha and gamma spectral frequencies contrast with the response of delta-theta and beta activities to NMDA receptor blockade as the reduction in current density with ketamine, particularly in the low frequencies, is contrary to the observation that increased local and global EEG delta/theta is one of the more consistent findings reported in unmedicated, first episode, and chronic SZ patients (Boutros et al., 2008; Moran and Hong, 2011; Kim et al., 2015).

Thus, acute NMDA receptor hypofunction may mediate some of the EEG disturbances associated with psychosis, particularly those relating to high frequency gamma perturbations, which in this study likely result from ketamine inhibition of NMDA receptors on GABA interneurons and disinhibition of pyramidal neurons (Grunze et al., 1996; Maccaferri and Dingledine, 2002; Jackson et al., 2004; Homayoun and Moghaddam, 2007) subpopulations, some of which act to increase cortical gamma during inhibition (Lovett-Barron et al., 2012; Royer et al., 2012). Considering ketamine’s multitude of effects, with actions at muscarinic, opioid, and adrenergic receptors and actions blocking serotonin and norepinephrine transporters (Bergman, 1999; Chen et al., 2009; Stahl, 2013), any one or more of these mechanisms [and/or the purported increase in non-NMDA glutamate (i.e., AMPA and kainate) receptor neurotransmission resulting from release of GABA restraint during impaired NMDA receptor function (Olney and Farber, 1995; Moghaddam et al., 1997)] may underly changes in non-gamma rhythms seen in SZ and with acute NMDA receptor blockade. Given that oxidative stress is thought to play a crucial role in several brain disorders including psychosis (Sorce and Krause, 2009; Schiavone et al., 2012, 2016), is involved in the modulation of neuronal activity (Infanger et al., 2006), and is increased with acute subanesthetic doses of ketamine and controls the resultant prefrontal glutamate release accompanying NMDA receptor antagonism (Sorce et al., 2010), brain oxidative stress may be one novel mechanism underlying the SZ-like electrophysiological changes with ketamine administration.

Contributing cortical sources to our observed ketamine modulated EEG profile were examined within the TN model of SZ. Although there is increasing interest in the pathophysiology of brain networks in SZ, to our knowledge this is the first human study to explore CSD in nodes of three well defined large-scale neural networks during acute NMDA receptor hypofunction. Directional changes in surface-level EEG current density induced by ketamine within each frequency band were mirrored by CSD alterations across the networks and, whereas only with alpha CSD were all three networks shown to be affected by NMDA receptor blockade, nodes of at least one or two of the networks evidenced ketamine-induced changes in delta (DMN), theta (DMN, CEN), beta (SN), and gamma (DMN, SN) rhythms. Activity of individual high and low frequencies is associated with both overlapped and distinct sensory and cognitive functions but given the general observation that reductions in low frequency and increases in high frequency rhythms are associated with increased arousal and behavioral activation (Moran and Hong, 2011), our frequency specific oscillatory changes with ketamine generally suggest that affected nodes within the respective large scale networks are *hyperactivated* during NMDA antagonist treatment. The exception is seen with the ACC, which showed alpha reductions and gamma increases (activation) along with decreases in beta (suppression) during ketamine administration. This general pattern of cortical activation during waking states is very similar to that reported in healthy volunteer studies utilizing fMRI and PET to image resting cerebral blood flow and brain metabolic response to ketamine’s blockade of NMDA receptors (Lahti et al., 1995; Breier et al., 1997; Vollenweider et al., 1997; Deakin et al., 2008; De Simoni et al., 2013; Doyle et al., 2013; Scherbinin et al., 2015). Although there are discrepancies in these neuroimaging studies, ketamine has typically activated frontal and temporal lobe structures as well as the AI and PCC but has suppressed the VMPFC while exhibiting mixed effects on the ACC, which has been found to be either hyperactive or hypoactive with acute antagonist treatment.

Accumulating neuroimaging evidence in healthy individuals indicates that an imbalance between the normally anti-correlated DMN (internal, self-related processing) and CEN (external environment/task-related processing) may underlie clinical and cognitive features of various psychiatric disorders (Menon, 2011), including SZ (Northoff, 2015). Although aberrant glutamatergic signaling in either the DMN or CEN may disrupt this balance, the AI-ACC nodes of the SN network, and the AI in particular, play a critical and causal role in the switch between activation and deactivation of the two large-scale brain networks (Sridharan et al., 2008). Structural and functional abnormalities across the different stages of SZ occur in the nodes of SN (Palaniyappan et al., 2012) and dopaminergic dysfunction in the SN is viewed as having a central role in aberrant motivational salience (Kapur, 2003) and clinical symptoms (Palaniyappan and Liddle, 2012) in individuals with psychosis. GABA/glutamate coupling is also thought to mediate SN functions and subanesthetic doses of ketamine have been specifically shown to increase glutamate turnover in the ACC, consistent with studies in SZ in which glutamatergic levels are elevated in the ACC of early-stage, drug naïve or drug-free patients (Merritt et al., 2013; Poels et al., 2014).

Our findings of ketamine-induced increases in gamma in the ACC and AI nodes, likely resulting from reduced excitation of GABAergic interneurons and the subsequent disinhibition of glutamatergic pyramidal neurons, may represent elevated noise at pyramidal cell assemblies (Moran et al., 2015), which may act to reduce high frequency signal-to-noise ratio in response to sensory input (Saunders et al., 2012) and disrupt information processing in these networks. Given that activity in the SN typically precedes and predicts activity in both DMN and CEN (Sridharan et al., 2008), implying that the SN coordinates multi-networking activity, then alterations in gamma in the SN may act to decrease the inherent anti-correlation between the DMN and CEN, as suggested by the co-activation of these networks, which is indexed by reductions in delta/theta and alpha activity. Decreases in anti-correlation between the DMN and CEN are thought to diminish the boundaries or distinction between internally and externally oriented cognitions, confusing internal and external mental contents and resulting in “self-environment blurring” (Vollenweider et al., 1997) – thus providing a mechanistic path that may explain several core symptoms of SZ.

Oscillations generated by thalamocortical circuits are thought to be responsible for the synchronization of neural activity between different cortical regions and, depending on the frequency range of the most prominent oscillation, are associated with the appearance of specific mental states. Generally, thalamocortically generated gamma is thought to be a potential explanation for coherence of perception in the brain while alpha serves to route information to downstream regions by inhibiting neuronal processing in task-irrelevant regions (Jensen and Mazaheri, 2010; Foxe and Snyder, 2011; Klimesch, 2012), thus allowing task-relevant regions to communicate. Accordingly, the reduction in alpha and increase in gamma seen in the SN with acute ketamine may be associated with reduced salience processing capabilities (due to impaired sensory/perceptual functions) and diminished registration of salience below the threshold that is needed to allow switching between the triple networks.

Study analysis of the subjective response to NMDA antagonist treatment assessed with CADSS showed significant dissociative effects, with ketamine infusion resulting in marked increases in amnesia, depersonalization, derealisation, and objective rater subscale scores. These dissociative phenomena, which have been consistently reported in previous ketamine studies with healthy volunteers and particularly in males (Morgan et al., 2006), are part of a spectrum of transient SZ-like psychoactive effects (including positive and negative symptoms) produced with acute subanesthetic ketamine (Krystal et al., 1994; Malhotra et al., 1996; Adler et al., 1998) and can be attenuated by inhibiting the reuptake glycine, a co-agonist at the strychnine-insensitive glycine-β site on the NMDA receptor (D’Souza et al., 2012).

Of the perceptual effects we observed with ketamine infusion, only changes in depersonalization scores were related to the EEG alterations. Increases in depersonalization ratings were associated with reductions in alpha current density across the scalp and in specific nodes of the DMN (bilateral PCC), CEN (right PPC), and SN (right AI). Altered self-other boundary, experienced as depersonalization (feeling detached as if one’s body is unreal), is one of several so-called “basic symptoms” of prodromal SZ (Maggini et al., 2002; Raballo, 2012). There is considerable overlap in midline cortical structures comprising the neural network implicated in self-specificity with those showing high resting state in the DMN, and while evidence suggests that the pregenual ACC is specifically involved in self-processing (Qin and Northoff, 2011), several neuroimaging studies have also pointed to the AI as being the representational cortex for the sense of self (Craig, 2002, 2009). Alpha rhythms are considered among the most important building blocks for functioning, association and communication in the brain (Basar, 2012; Basar and Guntekin, 2012; Bazanova and Vernon, 2014). Alpha is prominent in the EEG DMN profile (Chen et al., 2008), overlaps with resting-state networks identified in fMRI (Jann et al., 2007; Mantini et al., 2007), and in addition to being the main neural oscillation of self-agency (a person’s feeling that his action is generated by himself; Kang et al., 2015), correlates with both perception of self-related stimuli and resting state glutamate concentration in the pregenual ACC (Bai et al., 2016). Given this context, our findings with ketamine suggest that glutamatergic modulated alpha activity may be one mechanism underlying pathological self-processing in SZ and may serve as a viable target for novel treatment interventions.

### Limitations

The present results provide preliminary informative insight into the some of the electrocortical mechanisms influenced by blockade of NMDA receptors but limitations of the study must also be considered. First, ketamine is one of the most selective NMDA receptor antagonists available for human studies but it has secondary sites of action unrelated to these glutamatergic receptors and thus additional experiments with more selective NMDA receptor antagonists are needed to specifically associate our EEG findings to NMDA receptor hypofunction. Also, some of the non-specific behavioral effects associated with ketamine (e.g., drowsiness) may have compromised the study blind, and future studies may want to systematically document these behaviors to determine how these potential confounds may impact statistical analyses and data interpretation. Studies may also consider incorporating into their design an active placebo comparator drug such as midazolam, which has similar pharmacokinetic properties, rapid anesthetic and behavioral effects as ketamine. Further, acute NMDA antagonism reproduces many features of SZ but others (auditory hallucination) appear only after chronic administration (Javitt et al., 2012). The design of this study does not explicitly allow for longitudinal inferences, but different effects of short-and long-term exposure to NMDA antagonists have been demonstrated for varying neurotransmitter systems and cognitive domains (Bubeniková-Valesová et al., 2008). EEG studies with animal models of chronic ketamine users are required to profile the progression of these acute oscillatory changes. Second, EEG has relatively low spatial resolution compared to other imaging modalities such as fMRI. Future multimodal studies may wish to combine EEG recordings with concurrent fMRI or MEG recordings to further verify the sources of EEG markers. Moreover, it may be advantageous to implement ICA in the eLORETA software (eLORETA-ICA) as ICA decomposition of EEG data becomes more correct in localization and more robust to artifacts (Jonmohamadi et al., 2014) and filtered ICA time series of EEG correlates with BOLD time series in specific resting networks (Hiltunen et al., 2014). For high frequencies, which may be contaminated by muscle artifact, it would be useful to supplement this approach with electromyographic recordings and to apply canonical correlation analysis (CCA) as a blind source separation technique to remove broadband or electromyographic noise from single EEG epochs (De Clereq et al., 2006). These pre-processing strategies as well as more recently recommended state-of-the-art methodologies for analyzing high-frequency (gamma range) activity should be adopted in future ketamine-EEG research (Jobert et al., 2012; Nottage and Horder, 2015). As well, EEG assessments did not include measures of synchronization to index oscillatory coherence which may have been influenced by ketamine and may have provided insight into the pattern of aberrant regional and intra- and inter-network functional connectivity associated with NMDA receptor antagonism. Third, the dynamic TN hypothesis was not directly tested with experimental conditions that would behaviourally challenge each specific network function. Only dissociative symptoms were monitored and we did not include assessments of positive, negative, or cognitive symptoms, each of which may have been uniquely affected by ketamine infusion and differentially associated with oscillatory alterations resulting from NMDA receptor blockade. For example, beta reductions in SN ACC hub with ketamine were not associated with depersonalization ratings but, given that this oscillatory frequency is predominantly in attention tasks, its dysfunction with NMDA receptor blockade may be relevant to aberrant salience processing.

## Conclusion

In summary, resting-state EEG appears to be a useful and efficient method for investigating the neuropharmacology of altered brain rhythms suspected to underlie perceptual/dissociative symptoms of SZ. In the present study, sensor-level and surface projected neural network electrocortical activity was investigated in a NMDA receptor hypofunction model of SZ and intravenous ketamine was shown to partly mimic the aberrant EEG activity observed in SZ patients and to alter oscillations in large-scale resting-state networks implicated in psychosis. Neuroelectric changes correlated with the severity of dissociative symptoms induced by NMDA receptor antagonist treatment. Together, these findings provide additional information on how the modulation of the glutamatergic system may regulate brain electric activity, supporting its potential utility both as a biomarker of NMDA receptor dysfunction and a possible target for novel treatments in psychiatric disorders such as SZ which involve glutamatergic deficits.

## Author Contributions

Each of the authors participated in this research by contributing to the conception and design of the project (VK), participant screening (SdS, JC, DS, HB, and VI), performance of the experiment (SdS, JC, DS, HB, and JM), electrophysiology and statistical analysis (SdS), and interpretation (VK and SdS).

## Conflict of Interest Statement

The authors declare that the research was conducted in the absence of any commercial or financial relationships that could be construed as a potential conflict of interest.

## Footnotes

1 http://talairach.org/
